# Eco-functional deployment of indigenous nitrogen-fixing microbes to enable temperate crop cultivation in tropical climates

**DOI:** 10.1038/s41598-026-43406-x

**Published:** 2026-03-06

**Authors:** Shazwana Shaárani, Nurul Syazwani Ahmad Sabri, Fatimah Azizah Riyadi, Siti Noor Fitriah Azizan, Fazrena Nadia Md Akhir, Nor’azizi Othman, Hirofumi Hara

**Affiliations:** 1 Department of Chemical and Environmental Engineering, Malaysia-Japan International Institute of Technology, Universiti Teknologi Malaysia, Jalan Sultan Yahya Petra, Kuala Lumpur, 54100 Malaysia; 2 Universiti Utara Malaysia Kampus Kuala Lumpur, Jalan Raja Muda Abdul Aziz, Kampung Baru, Wilayah Persekuatuan Kuala Lumpur, 50300 Malaysia; 3 Department of Chemical Engineering, Faculty of Engineering, Universitas Indonesia, Depok, 16424 West Java Indonesia; 4 School of Agriculture, Meiji University, 1-1-1 Higashi-Mita, Tama-ku, Kawasaki, 214-8571 Japan; 5 Department of Mechanical Precision Engineering, Malaysia-Japan International Institute of Technology, Universiti Teknologi Malaysia, Jalan Sultan Yahya Petra, Kuala Lumpur, 54100 Malaysia; 6 Department of Biotechnology, Graduate School of Agricultural and Life Sciences, The University of Tokyo, 1-1-1, Yayoi, Bunkyo-ku, Tokyo, 113-8657 Japan

**Keywords:** Climate change, Plant growth-promoting rhizobacteria, Putative diazotrophic traits, Soil cooling, Tropical agriculture, Ecology, Ecology, Environmental sciences, Microbiology, Plant sciences

## Abstract

Climate change and increasing food demand pose major challenges to agricultural productivity, particularly for temperate crops cultivated under tropical conditions. This study investigated whether indigenous bacterial isolates obtained from cooled tropical soils exhibit putative diazotrophic traits and promote the growth of lettuce, a representative temperate crop, under tropical greenhouse conditions without soil cooling. Seven bacterial isolates were initially screened using preliminary assays indicative of putative diazotrophic traits, including nitrogen-free semisolid medium, bromothymol blue assays, and PCR detection of *nif*-related genes. Two strains, *Agromyces* sp. C10 and *Bacillus* sp. C21, were selected for further evaluation. Inoculation with these strains enhanced lettuce biomass in the presence and absence of fertilizer and was associated with increased total soil nitrogen after harvest. Although these results do not provide direct functional evidence of biological nitrogen fixation, they indicate that indigenous bacteria from cooled soils may serve as promising plant growth-promoting candidates for temperate crop cultivation in tropical environments.

**Supplementary Information:**

The online version contains supplementary material available at 10.1038/s41598-026-43406-x.

## Introduction

Climate change is widely acknowledged to impact agricultural production, crops, and food security on a global scale. According to the Intergovernmental Panel on Climate Change (IPCC)^1^, extreme weather events will likely have a disruptive effect on global food supplies and cause food prices to rise. This situation is further compounded by increases in temperature, precipitation, and atmospheric CO_2_ levels. These increases can induce changes in plant tissues at the morphological, anatomical, physiological, and biochemical levels, affecting the growth and development of various plant organs^2,3^. Among the most affected are temperate crops, which typically require cooler soil and air temperatures to thrive. As global food demand rises, there is a pressing need to explore sustainable strategies that enable the cultivation of high-value temperate crops in tropical environments. These challenges highlight the need for alternative, low-input strategies to support temperate crop cultivation under tropical conditions by harnessing indigenous soil microorganisms and environmentally mediated microbial selection processes.

Plant growth-promoting rhizobacteria (PGPR) are resilient soil microorganisms that offer an eco-friendly method for preserving and improving the soil’s nitrogen availability. Many initiatives are being undertaken to create new strains that have beneficial agronomic traits and enhance the effectiveness and dependability of microbial-based products for large-scale farming operations^4^. Temperate crops, such as lettuce and radish, are adapted to cooler climates and are particularly sensitive to elevated temperatures. Exposure to tropical conditions can lead to reduced germination rates, impaired photosynthesis, and premature bolting, ultimately affecting yield and quality^5,6^. These physiological constraints limit their cultivation in tropical lowlands without environmental modification. For many years, plant growth-promoting microbes (PGPMs), such as rhizobia and mycorrhizae, have been shown to enhance plant development under both stressed and non-stressed conditions. The use of microbial inoculants has become increasingly common in recent decades. It is advisable to isolate PGPMs from their native environments, propagate them under controlled conditions, and then reintroduce them as microbial inoculants to enhance their effectiveness in promoting plant growth. This is because although PGPMs are naturally present in the rhizosphere and plant tissue, their populations may be insufficient to produce the desired effects^7,8^. Certain symbiotic and free-living bacteria can fix atmospheric nitrogen into plant-usable forms, such as ammonium, through biological nitrogen fixation^9^. Both legumes and non-leguminous plants can participate in symbiotic nitrogen fixation, although legumes have been studied more extensively than non-leguminous plants^8^.

Components of nitrogenase and other regulatory proteins are encoded by nitrogen fixation genes (also known as *nif* genes). To convert dinitrogen into ammonia, the metalloenzyme complex known as nitrogenase is required to catalyze the ATP-dependent reduction of dinitrogen (nitrogen fixation)^10^. Among the most well-studied genes for nitrogen-fixing bacteria are those encoding the nitrogenase enzyme complex. The *nifH* gene encodes the iron protein subunit of nitrogenase, which facilitates the transfer of electrons during nitrogen fixation. Complementing *nifH* are the *nifD* and *nifK* genes, responsible for encoding the molybdenum-iron protein subunits that carry out the catalytic reduction of nitrogen gas to ammonia. Additionally, genes such as *nifE*, *nifN*, and *nifX* are crucial for the synthesis and insertion of the essential iron-molybdenum cofactor into the molybdenum-iron protein. The *nifH* gene, which encodes the iron nitrogenase unit of the nitrogenase complex, is favored for this purpose because of its well-conserved amino acid sequence and the availability of comprehensive reference sequence databases^11,12^. The information obtained from the expression of the *nif* gene and the activity of nitrogenase in non-legume diazotrophs has offered a snapshot of the various plant contexts, such as the plant’s growth stage and circadian rhythm depending on the variability of plant-based carbon sources^13^. Variables such as host, bacterial strains, temperature, light, soil pH, soil chemical and physical properties, and interactions with other microorganisms all might influence plant development.

Soil cooling was applied in this study as an ecological filtering strategy to alter microbial community composition and enrich for bacteria capable of maintaining metabolic activity under lower-temperature conditions. Temperature is a key environmental factor shaping soil microbial structure and function, influencing microbial growth rates, community composition, and nutrient cycling processes^14,15^. Previous studies have shown that reduced temperature can shift microbial communities toward stress-tolerant or slow-growing taxa and alter nitrogen transformation pathways, including mineralization and immobilization dynamics^16^. In our previous soil-cooling trials, we observed an increased frequency of bacterial isolates exhibiting diazotroph-like growth characteristics, which motivated their further screening in the present study. Although soil cooling itself was not applied during the greenhouse cultivation experiment, it served as a pre-selection tool to obtain indigenous bacterial candidates with potential functional relevance for temperate crop cultivation under tropical conditions. Lettuce (*Lactuca sativa*) was selected as a model temperate crop because it is highly sensitive to temperature stress and nitrogen availability and is widely used as an indicator species in controlled-environment agriculture and plant-microbe interaction studies. Its short growth cycle, shallow root system, and strong responsiveness to nitrogen inputs make it suitable for evaluating preliminary plant growth-promoting effects under tropical greenhouse conditions. Although lettuce represents only one temperate crop system, it provides a tractable and well-characterized model for assessing whether indigenous bacterial isolates can enhance temperate crop growth under tropical conditions. The results obtained from this system are intended to provide proof-of-concept evidence that can guide future validation in additional temperate crop species. Therefore, the objective of this study was to isolate indigenous bacterial strains from cooled soil that exhibit characteristics consistent with nitrogen-fixing potential and to evaluate their plant growth-promoting effects on lettuce, a representative temperate crop, under tropical greenhouse conditions without the use of a soil cooling system.

## Results

### Isolation of putative diazotrophic bacteria from cooled soil

Soil samples used for isolating potential nitrogen-fixing microbes were obtained from previously cooled soil, as described in our earlier study by Sha’arani et al.^17^. This soil had shown higher nitrogen content compared to uncooled soil under tropical conditions following temperate crop cultivation^17^. After the isolation steps, seven candidates were obtained as putative diazotrophic bacteria based on preliminary screening assay, which exhibited positive reactions on N-free semisolid medium, a differentiating medium for preliminary screening of PGPR for putative diazotrophic traits^18^. Initial screening for putative diazotrophic traits was conducted using N-free medium. Notably, the isolates demonstrated differential growth responses depending on the carbon source provided in the nitrogen-free medium (mannitol, sucrose, or malate) highlighting variability in their carbon utilization preferences. Preliminary PCR screening also supported the presence of *nifF* and *nifD* genes in the isolates, which was later confirmed by genome annotation. Based on screening results, strains C10 and C21 were selected for further study due to their consistent growth on nitrogen-free media, positive bromothymol blue color change (see Supplementary Fig. S1 and Supplementary Fig. S2 online) and successful amplication of genes annotated as nitrogen fixation-related genes with unknown or putative functions.

### Bioinformatics analysis of draft genome sequence of *Agromyces* sp. C10 and *Bacillus* sp. C21

Draft-genome sequencing and annotation results were further analyzed to provide useful insights into the processes that drive plant growth (*Agromyces* sp. C10 and *Bacillus* sp. C21)^19^. Genes annotated as *nifF*,* nifD*,* glnR*, and components of the *anf* and *vnf* complexes were identified in the draft genomes of *Agromyces* sp. C10 and *Bacillus* sp. C21 (Supplementary Table S1). Despite the identification of nif-, anf-, and vnf-like genes, the low sequence similarity of these genes to previously characterized homologs does not provide evidence for a complete or functional nitrogenase system. These findings are consistent with earlier PCR-based screening and are indicative of putative diazotrophic traits; however, they do not constitute functional proof of biological nitrogen fixation. The draft genome of *Agromyces* sp. C10 revealed the presence of the nitrogen metabolism regulator *glnR*, a member of the OmpR family. This gene in *Agromyces* sp. C10 had 62% identity (bit score: 269) with previously reported homologous genes. The LysR family was found in both strains, *Agromyces* sp. C10 and *Bacillus* sp. C21. We also observed the *nod* gene in *Agromyces* sp. C10. The *nodD* genes *nodD1*, *nodD2* and *nodD3* encode LysR family transcriptional regulators commonly associated with the regulation of nodulation genes in symbiotic nitrogen-fixing bacteria^20,21^. The detection of a nodD-like gene in *Agromyces* sp. C10 should be interpreted with caution, as this genus is not known to form symbiotic nodules and no experimental evidence of nodulation or symbiotic interaction was obtained. Therefore, this finding does not support conclusions regarding symbiotic nitrogen fixation. Functional categorization of the genes presents in *Agromyces* sp. C10 and *Bacillus* sp. C21 based on COG is shown in Supplementary Table S2 and Supplementary Table S3, respectively. The protein-coding genes from the draft genome sequence of *Agromyces* sp. C10 were annotated using eggNOG-mapper and classified into 20 functional classes/subsystems according to COG. Based on the COG functional categories, general processes and metabolic pathways related to amino acids, carbohydrates, fatty acids, and lipids were dominant. Annotated genes related to nitrogen metabolism and putative diazotrophic traits in *Agromyces* sp. C10 and *Bacillus* sp. C21 are tabulated in Supplementary Table S4 and Supplementary Table S5, respectively. The draft genome assemblies do not demonstrate the presence of a complete or functional nitrogenase gene cluster, and the low sequence similarity of annotated nif-, anf-, and vnf-like genes does not support claims of pathway novelty or nitrogen fixation activity. Therefore, no inference regarding functional nitrogen fixation or novel nitrogen fixation pathways can be made based on these genomic data alone.

### Evaluation of putative diazotrophic traits and support of temperate crop growth under a lowland tropical climate

To evaluate the ability of the isolates to support temperate crop cultivation under a tropical climate without soil cooling, lettuce was chosen as a representative temperate root crop, similar to a previous study^22,23^. *Agromyces* sp. C10 and *Bacillus* sp. C21 had a significant positive effect on the growth of lettuce in terms of weight, height and root length compared to the control (without inoculant and fertilizer), as depicted in Table 1 and Supplementary Fig. S3 online (lane 1; without fertilizer, lane 3; *Agromyces* sp. C10 inoculation, lane 5; *Bacillus* sp. C21 inoculation). Inoculation with *Agromyces* sp. C10 and *Bacillus* sp. C21 was associated with enhanced lettuce growth under tropical greenhouse conditions. These effects were correlated with increased lettuce biomass and root development, as shown in Table 2 and Supplementary Fig. S3 online (lane 2; with fertilizer, lane 4; *Agromyces* sp. C10 inoculation with fertilizer, lane 5; *Bacillus* sp. C21 inoculation with fertilizer).

**Table 1:** Effect of nitrogen-fixing bacteria on lettuce growth parameters without fertilizer (*n* = 3).

	Weight (g)	Height (cm)	No. of leaves	Length of root (cm)
Control(without fertilizer)	2.93 ± 0.47^c^	10.1 ± 1.3^c^	5^c^	6.0 ± 1.1^c^
*Agromyces* sp. C10	13.10 ± 0.93^a^	13.9 ± 0.7^a^	8^a^	7.2 ± 1.6^a^
*Bacillus* sp. C21	12.82 ± 0.42^a^	18.8 ± 0.8^a^	6^a^	8.3 ± 1.1^a^

Values represent mean ± SD (*n* = 3). Different lowercase letters indicate significant differences among treatments according to one-way ANOVA followed by Tukey’s HSD test (*p* < 0.05).

**Table 2:** Effect of nitrogen-fixing bacteria on lettuce growth parameters with fertilizer (*n* = 3).

	Weight (g)	Height (cm)	No. of leaves	Length of root (cm)
Fertilizer	13.67 ± 0.22^b^	16.0 ± 0.8^b^	9^b^	8.1 ± 0.6^b^
Fertilizer with *Agromyces* sp. C10	14.47 ± 0.44^a^	15.9 ± 0.9^b^	9^b^	8.1 ± 0.8^b^
Fertilizer with *Bacillus* sp. C21	17.81 ± 0.52^a^	23.6 ± 1.3^a^	10^a^	8.7 ± 0.9^a^

Values represent mean ± SD (*n* = 3). Different lowercase letters indicate significant differences among treatments according to one-way ANOVA followed by Tukey’s HSD test (*p* < 0.05).

### Total nitrogen content in soils after growth of temperate crop supplying *Agromyces* sp. C10 and *Bacillus* sp. C21

To determine the ability to maintain nitrogen in soils after the growth of lettuce under a tropical climate, soil samples were collected, and the nitrogen content was measured. The total nitrogen content was discovered to be significantly higher in both *Agromyces* sp. C10 and *Bacillus* sp. C21 compared to the control (without inoculant and fertilizer), as tabulated in Table 3. Meanwhile, when compared with fertilizer, both isolates led to increased nitrogen content, as shown in Table 4. These results indicated that *Agromyces* sp. C10 and *Bacillus* sp. C21 were associated with increased total soil nitrogen content after harvest.

**Table 3:** Total nitrogen of soil after harvesting without fertilizer (*n* = 3).

	Nitrogen content (mg/kg)
Control (no nitrogen fertilizer)	11,400 ± 283^c^
Inoculation of *Agromyces* sp. C10	14,533 ± 531^a^
Inoculation of *Bacillus* sp. C21	15,900 ± 1,042^a^

Values represent mean ± SD (*n* = 3). Different lowercase letters indicate significant differences among treatments according to one-way ANOVA followed by Tukey’s HSD test (*p* < 0.05).

**Table 4:** Total nitrogen of soil after harvesting with fertilizer (*n* = 3).

	Nitrogen content (mg/kg)
Addition of fertilizer	15,033 ± 1,821^b^
Fertilizer with the inoculation of *Agromyces* sp. C10	15,167 ± 1,613^b^
Fertilizer with the inoculation of *Bacillus* sp. C21	16,633 ± 419^a^

Values represent mean ± SD (*n* = 3). Different lowercase letters indicate significant differences among treatments according to one-way ANOVA followed by Tukey’s HSD test (*p* < 0.05).

## Discussion

This study successfully isolated seven bacterial strains exhibiting putative diazotrophic traits from cooled soil, all of which showed positive growth on nitrogen-free media. Consistent with Kifle and Laing^24^, the isolates utilized sucrose effectively and showed metabolic activity associated with nitrogen-related processes (Figure S1). Their ability to shift bromothymol blue from yellow to blue is consistent with putative diazotrophic traits. The increase in pH caused by bacterial metabolic activity in nitrogen-free medium may reflect nitrogen-related metabolic processes but cannot be attributed specifically to biological nitrogen fixation. Furthermore, the effectiveness of these two selected strains in stimulating the growth of temperate crops, notably lettuce, under tropical climate conditions was investigated. *Agromyces* sp. C10 and *Bacillus* sp. C21 alone were associated with enhanced crop development. The findings showed that the treatment had a considerable positive impact on a variety of growth traits, including plant weight, height, leaf count, and root length. These findings suggest that *Agromyces* sp. C10 and *Bacillus* sp. C21 are promising plant growth-promoting candidates for temperate crops under tropical conditions. In addition, the effect of these strains on total soil nitrogen content was evaluated by measuring the total nitrogen content in the soil following lettuce growing. The considerable increase in total nitrogen content is consistent with bacterial inoculation effects on soil nitrogen dynamics but does not allow inference of biological nitrogen fixation. Growth on nitrogen-free semisolid media, bromothymol blue color change, and partial PCR detection of nitrogen fixation–related genes are commonly used for preliminary screening of putative diazotrophs; however, these approaches do not constitute functional proof of biological nitrogen fixation. Such assays cannot distinguish between true N₂ fixation and alternative metabolic strategies, including utilization of trace nitrogen sources, intracellular nitrogen recycling, or altered nitrogen mineralization dynamics. Similarly, although bacterial inoculation was associated with increased total soil nitrogen after harvest, total nitrogen measurements alone do not distinguish newly fixed atmospheric nitrogen from nitrogen derived from residual soil pools, microbial biomass accumulation, or altered mineralization processes. Without direct measurements of nitrogenase activity or isotopic tracing (e.g., ¹⁵N-based assays), the specific contribution of biological nitrogen fixation cannot be determined. Moreover, the enhancement of lettuce growth observed in this study may also involve other plant growth–promoting mechanisms, such as phytohormone production, phosphate solubilization, siderophore production, or modulation of the rhizosphere microbiome, which were not evaluated and therefore cannot be excluded. Although the isolates demonstrated consistent growth on nitrogen-free media and genomic screening revealed the presence of *nif*-related genes, their nitrogen-fixing capability has not yet been validated through quantitative assays. Serial passaging on nitrogen-free medium was performed to ensure phenotype stability; however, direct measurements such as the acetylene reduction assay (ARA) analyses were not conducted in this study. Therefore, the current evidence supports putative diazotrophic traits, and further functional validation will be necessary. A further consideration is the absence of an equal-nitrogen control in which non-inoculated plants receive the same amount of plant-available nitrogen as the inoculated group. Including this control in future studies will allow a clearer distinction between growth responses driven by microbial nitrogen contribution and those arising from other beneficial traits of the isolates. While the current results point to possible nitrogen-related contributions, an equal-nitrogen comparison will help determine the extent to which nitrogen supply contributes to the overall plant growth improvement relative to other plant-growth-promoting mechanisms. To fully evaluate the potential of these isolates, it is crucial to test them in non-sterilized field soil, where natural microbial communities and environmental conditions will provide a more comprehensive understanding of their applicability in real-world agricultural settings. Small-scale trials in unsterilized soil will enable us to assess the consistency of their effects and further validate their role in promoting crop growth in complex, dynamic environments. Together, these findings provide a preliminary basis for identifying *Agromyces* sp. C10 and *Bacillus* sp. C21 as promising candidates for sustainable crop enhancement in tropical environments, and they justify further targeted investigations into their putative diazotrophic traits and associated plant-growth-promoting mechanisms.

Temperate crop cultivation in lowland tropical regions holds significant importance for several reasons. It presents a chance for the diversification of agricultural methods^25^. The successful growth of lettuce under tropical conditions without soil cooling, as demonstrated in this study, highlights the potential for expanding temperate crop cultivation in lowland tropical regions. This approach could contribute to agricultural diversification and food security, especially in areas where highland cultivation is limited. This diversification can help improve food security by lowering reliance on a small number of crops and establishing a more resilient agricultural system^26^. Temperate crops like lettuce, grapes, and strawberries are in high demand and have a high economic value^5,27,28^. Furthermore, temperate crops have inherent features that make them ideal for production in tropical environments. They frequently show more adaptability to changing climatic conditions, such as fluctuations in temperature and photoperiod. This adaptation enables temperate crops to endure the challenges offered by tropical climes, such as higher temperatures, humidity, and disease pressures^29^. The production of temperate crops can also be explored in lowland areas due to the restricted availability of highland regions in tropical climates. Lowland tropical climates frequently have larger agricultural land tracts suitable for agriculture^30^. By cultivating temperate crops in lowland areas, farmers can continue to reap the benefits of agricultural diversification, nutritional value, economic opportunities, and climate change adaptation.

Genes and enzymes associated with nitrogen fixation play a crucial role in the biological process of converting atmospheric nitrogen (N_2_) into a form that plants can utilize. Previous studies have reported the presence of alternative nitrogen fixation pathways in certain bacteria^31–33^. For example, some bacteria utilize alternative nitrogenase enzymes, such as a*nf* and *vnf* complexes, to fix nitrogen under specific conditions^34^. *Anf* and *vnf* complexes are similar to Mo-nitrogenase in their ability to fix nitrogen, but they have different structural and catalytic properties. In this study, the presence and characterization of nitrogen fixation genes and enzymes in the isolated diazotrophic bacteria, *Agromyces* sp. C10 and *Bacillus* sp. C21, were investigated. It is noteworthy to mention that despite the low similarity of nitrogen-fixing genes such as *nifF*, *nifD*, *anf*, and *vnf* complex, in the isolates in this study, *Agromyces* sp. C10 and *Bacillus* sp. C21 exhibited putative diazotrophic traits (Table S5). Surprisingly, the analysis showed that some genes in *Agromyces* sp. C10 demonstrated high homology and similarity with genes encoding *vnf* in the reference genome, with 56% similarity (bit score: 239) to the *vnf* of *Azotobacter chroococcum* mcd 1 (UniProt Entry: P06118), while *Bacillus* sp. C21 demonstrated high homology and similarity with genes encoding *glnR* in the reference genome, with 100% similarity (bit score: 277) to the *glnR* of *Bacillus subtilis* (strain 168) (UniProt Entry: P37582). Polyphosphate kinase (EC 2.7.4.1) surrounds this regulatory gene in *Agromyces* sp. C10. It has been observed that polyP levels can increase during nitrogen-related metabolic processes, and PPK may play a role in regulating polyP synthesis under such conditions^35^. Meanwhile, *Bacillus* sp. C21 contains glutamine synthetase type I (EC 6.3.1.2). Glutamine synthetase (GS) (EC 6.3.1.2) is an essential enzyme of nitrogen metabolism found in all domains of life. Using ATP as energy, it catalyzes the production of glutamine from glutamate and ammonia^36^. These findings cannot be interpreted as evidence of alternative or unique nitrogen fixation mechanisms. Research into these organisms is warranted because of their putative diazotrophic traits, plant growth-promoting potential, and distinctive genetic composition.

In addition, bioinformatics analysis of the draft genome sequences of *Agromyces* sp. C10 and *Bacillus* sp. C21 revealed the presence of genes involved in nitrogen metabolism regulation. For instance, the *glnR* gene, an encoding OmpR-type response regulator, is present in *Agromyces* sp. C10, indicating that it has the potential to be used to regulate genes involved in nitrogen metabolism. Both *Agromyces* sp. C10 and *Bacillus* sp. C21 displayed adequate homologous sequences, which may have contributed to the activity observed in both strains (Table S5). Similarly, the *glnR* gene in *Bacillus* sp. C21 implies that it regulates gene expression in nitrogen fixation/metabolism. Previously, this gene was reported to be involved in the regulation of nitrogen metabolism in certain bacteria, such as *Bacillus subtilis*, by controlling the expression of glutamine synthetase (GS) along with multiple genes associated with nitrogen metabolism^37^. A GlnR homolog from *Staphylococcus aureus* was found to complement a *B. subtilis glnR* mutant, regulating GS levels and *glnRA* expression in a nitrogen-dependent manner^37^. These findings highlight the potential regulatory mechanisms that these bacteria employ to control nitrogen metabolism pathways. The regulation of nitrogen fixation by ammonia in gram-negative diazotrophs is well characterized^38^ yet not in gram-positive bacteria. Nitrogen metabolism in *Bacillus* and other gram-positive bacteria is controlled globally by GlnR, a transcription regulator^38^. In *Bacillus subtilis*, GlnR functions as a repressor, inhibiting the expression of the *glnRA* operon when nitrogen levels are high. The inoculated plants’ overall phenotype compared to nitrogen-fertilized plants may suggest that apart from potential nitrogen-related metabolic effects, there might be other benefits for the host, such as the secretion of phytohormones and relieving the plants from abiotic stress^39,40^. It is noteworthy that *Bacillus* sp. C21 exhibited a significant resemblance and similarity to genes encoding GlnR in the reference genome, displaying 100% similarity (bit score: 277).

There are promising results for lettuce growth with and without fertilizer after inoculation with *Agromyces* sp. C10 and *Bacillus* sp. C21. According to Souza and Tavarez^41^, this increase in plant height is directly tied to the functions of N in cell division, plant metabolism, and photosynthetic processes. Inoculation with plant growth-promoting bacteria significantly increased lettuce height, indicating their potential to enhance plant growth under nitrogen-limited conditions. Similar results for PGPR inoculation have been reported for different crops, such as chili, cucumber, tomato and legumes^42–46^- all of which are well-adapted to tropical environments. However, reports describing PGPRs isolated specifically from tropical environments to support temperate crop cultivation remain limited. It is worth noting that *Agromyces* sp. C10 itself can support lettuce without fertilizer. *Agromyces* sp. C10 was associated with changes in soil nitrogen dynamics and plant nitrogen status under tropical conditions, potentially supporting the growth of temperate crops without the need for soil cooling, similar to the role played by some PGPR. This observation aligns with its genomic profile, which revealed the presence of nitrogenase-related genes such as *nifF*,* nifD*,* anf*, and the *vnf* complex, albeit with low sequence similarity to known counterparts. Currently, there is limited information available on the putative diazotrophic activity of *Agromyces* species. Studies by Gtari et al.^47^ and Sellstedt and Richau^48^ have reported the presence of nitrogenase genes in certain strains of *Agromyces*, suggesting that they may exhibit putativediazotrophic traits. In this study, the nitrogenase genes *nifF*, *nifD*, *anf*, and the *vnf* complex (as shown in Table S5) were present in *Agromyces* sp. C10, albeit with low similarity. Further investigation is required to validate this observation and to identify the specific environmental or physiological conditions that enable nitrogen fixation in *Agromyces.*

On the other hand, it has been noted that a number of *Bacillus* species have been reported to exhibit diazotrophic traits or nitrogen-fixation potential^49–52^. The ability to fix nitrogen was demonstrated by *Bacillus* species from the soil of *Arabidopsis* plants, including *Bacillus amyloliquefaciens* and *Bacillus velezensis*^53^. The benefits of the interactions between nitrogen-fixation and plants in the family *Bacillus*, such as the bulbs of *Lilium wardii*.^54^, rice^55,56^, potato^57^ and maize^58^, have already been reported. *Bacillus* species are important PGPR and endophytes that were identified as producers of growth-promoting substances, bioactive chemicals, and metabolites with antimicrobial effects^59^. The results of this study demonstrated the beneficial effects of *Bacillus* sp. C21 inoculation on lettuce growth. Lettuce inoculated with *Bacillus* sp. C21 showed increased plant height and weight compared with the control, which was in line with the results of previous studies^24^. It has been demonstrated that strains of the genus *Bacillus* work well as biofertilizers and biocontrol agents in supporting agricultural development because they possess a variety of features that promote plant growth^60,61^. Although *Bacillus* species exhibiting diazotrophic traits or nitrogen-fixation potential have been reported, their isolation from artificially cooled soil used for temperate crop cultivation in tropical climates suggests a potentially underexplored ecological niche that warrants further investigation. To our knowledge, reports describing the isolation of Bacillus strains from artificially cooled soils used for temperate crop cultivation under tropical conditions remain limited, in part because of the complexity of genetically transforming these bacteria, highlighting the need for further investigation in this ecological context. Therefore, it is important to study the putative diazotrophic traits and plant growth-promoting mechanisms of each Bacillus and Agromyces strain individually to determine their potential for use in agricultural and environmental applications.

Temperate crop cultivation in tropical regions presents numerous challenges, particularly in the context of climate change. Rising temperatures, shifting rainfall patterns, and an increase in the frequency of extreme weather events all pose substantial threats to the productivity and sustainability of temperate crops in these regions. However, this study’s findings offer promising solutions and potential applications for addressing these challenges. Moreover, microbial inoculants may contribute to improving soil health and resilience under changing climatic conditions. Climate change may affect soil characteristics, resulting in lower soil fertility, increased erosive processes, and reduced water-holding capacity. The use of microbial inoculants can increase soil organic matter content, nutrient cycling, and microbial diversity, thereby enhancing soil structure and resilience. This process, in turn, improves the soil’s ability to retain moisture, withstand extreme weather events, and promote plant growth even in challenging conditions. The outcomes showed that, even in tropical conditions without soil cooling, these strains have a favorable impact on the growth of lettuce, a representative temperate crop. The integration of microbial inoculants exhibiting putative diazotrophic traits may contribute to improving crop resilience and soil health under tropical greenhouse conditions.

## Materials and methods

### Isolation procedures of candidates for nitrogen fixation

All microbiological, molecular, and genomic analyses in this study were conducted as preliminary screening approaches to identify bacteria exhibiting putative diazotrophic traits and plant growth–promoting potential and were not intended to provide direct functional evidence of biological nitrogen fixation. Potential nitrogen-fixing bacteria were isolated from the cooled soil of harvested temperate root crops (radish) grown under a tropical climate^17^. A modified protocol (N-free semisolid media) was performed for isolation purposes^62^. Serial dilution was carried out to obtain pure cultures of diazotrophic bacteria, which were then plated onto N-free (NF) plates containing either 20 g l^− 1^ mannitol, sucrose, or malate as the carbon source; 0.2 g/L K_2_HPO_4_; 0.2 g/L NaCl; 0.2 g/L MgSO_4_.7H_2_O; 0.1 g/L K_2_SO_4_; 5.0 g/L CaCO_3_; and 20 g/L agar (Merck) for solid agar media or 5 g/L of agar for semisolid media. Ten grams of soil sample (collected 15 cm below the soil surface) was transferred to a 250 mL Erlenmeyer flask containing 90 mL of sterile distilled water and shaken at 150 rpm in an orbital shaker incubator for 30 min. Plates with NF medium containing a carbon source for diazotrophic bacteria were then inoculated with 0.1 mL of the aforementioned suspensions (three replicates per dilution). The pH of the NF medium was adjusted to 6.5 using 98% H_2_SO_4_ and 50% NaOH. The inoculated NF medium plates were incubated at 30 °C for 5 days to reflect tropical temperature conditions. After incubation, colonies were transferred to fresh NF media, and two days later, they were streaked onto tryptone soy agar (TSA; Merck) plates. Bacterial isolates were chosen based on the size and shape of their colonies and their potential to grow on N-free media. These colonies were then subcultured onto TSA, heated to 30 °C in an incubator, purified, and kept at a temperature of -80 °C in 15% glycerol. Seven bacterial isolates were initially obtained based on growth on nitrogen-free semisolid medium. These isolates were subsequently screened using bromothymol blue assays and PCR amplification of nitrogen fixation-related genes. Strains C10 and C21 were selected for further analysis based on (i) reproducible growth on nitrogen-free media across repeated screenings, (ii) consistent bromothymol blue color change, and (iii) positive amplification of at least one nitrogen fixation-related gene. Growth behavior alone was not used as the sole criterion for selection. Putative diazotrophic traits were qualitatively screened by allowing the seven strains to grow in nitrogen-free bromothymol blue medium (sucrose: 10 g/L K_2_HPO_4_; 0.6 g/L MgSO_4_; 0.2 g/L K_2_SO_4_; 0.2 g/L NaCl; 0.1 g/L CaCO_3_, pH 6.8, 2.0 ml of 0.5% bromothymol blue solution).

A change in medium color from yellow to blue was used as an indicator of potential nitrogen-related metabolic activity. PCR amplification of nitrogen fixation–related genes (*nifH*,* nifD*, and *nifK*) was performed as a preliminary screening approach using published primer sets (Supplementary Table S6). Primer pairs PolF/PolR were used for *nifH*^63^, *nifD*-up/*nifD*-do for *nifD*^64^, and *nifHD*-f/*nifD*-r for *nifK*^65^. PCR reactions were carried out in a final volume of 20 µL containing genomic DNA, primers, dNTPs, reaction buffer, and Taq DNA polymerase. Amplification was performed in a PCR thermal cycler (Applied Biosystems) with the program as in Supplementary Table S6. Reactions without template DNA were included as negative controls. Primer sequences, annealing temperature, and references are provided in Supplementary Table S6. These PCR assays were used solely for screening of putative diazotrophic traits and were not intended to demonstrate functional biological nitrogen fixation.

### Draft genome sequencing analysis of isolates

Two out of seven strains exhibiting putative diazotrophic traits were further evaluated by draft genome sequencing. Approximately 1 Gb of paired-end data (2 × 150 bp) was generated by sequencing each sample on a NovaSeq 6000 platform (Illumina, San Diego, CA). The genome assembly was performed with Unicycler v0.4.8^66^. Genome assembly statistics were generated using QUAST^67^. Identified ribosomal RNA contigs were extracted using barrnap and compiled into a fasta file for traditional BLAST analysis with the GTDB 16 S rRNA database. Genomic features were annotated with NCBI PGAP v5.1^68^ and RAST v2.0^69^. Genes related to nitrogen fixation were identified using the KEGG database and a BLASTP search. Protein-coding genes derived from draft genome sequencing of strains C10 and C21 were annotated and classified according to functional class and subsystem categories based on COG and RAST. The raw data of draft genome sequence for *Agromyces* sp. C10 and *Bacillus* sp. C21 were deposited at GenBank under BioProject accession number *PRJNA825673*. The BioSample accession numbers for *Agromyces* sp. strain C10 and *Bacillus* sp. strain C21 are *SAMN27553938* and *SAMN27553940*, respectively. The GenBank accession numbers are *JALPSX000000000* (*Agromyces* sp. strain C10), and *JALPSZ000000000* (*Bacillus* sp. strain C21). Because draft genome assemblies were used, the presence, completeness, and functionality of nitrogen fixation–related gene clusters could not be conclusively determined. Gene annotations were interpreted conservatively, and low sequence similarity to known homologs was not considered evidence of novel nitrogen fixation pathways. Potential annotation artifacts and incomplete assemblies cannot be excluded.

### Experimental design for the growth of lettuce under a tropical climate

Commercial lettuce (*Lactuca sativa* L.) seeds were obtained from a local supplier (TRIO, Trio Garden Supplies Sdn. Bhd, Malaysia). Lettuce was selected as a representative temperate crop due to its sensitivity to temperature and nutrient stress and its frequent use as a model species in controlled-environment and microbial inoculation studies. Seeds were soaked in distilled water for two days to break dormancy, then surface-sterilized in 70% ethanol for 2 min and 2.5% sodium hypochlorite for 1 min prior to rinsing with 500 mL distilled water 4 times. Seven-day-old seedlings were transplanted into sterilized soil contained in 2-liter plastic pots (one seedling per pot). Each experiment was performed in triplicate. All growth measurements (weight, height, number of leaves, and root length) were obtained from three biological replicates (*n* = 3). The experimental setup consisted of 4 conditions: (i) with nitrogen fertilizers (PETRONAS Agrenas; 46% nitrogen); (ii) with nitrogen fertilizer together with inoculation of bacteria; (iii) without nitrogen fertilizer; and (iv) without nitrogen fertilizer and with inoculation of bacteria. To evaluate the association between bacterial inoculation and plant growth performance under tropical greenhouse conditions, lettuce was grown in 2-liter plastic pots filled with sterilized soil. The use of sterilized soil minimized background microbial variability but limits direct extrapolation of the results to non-sterile field conditions. The experiment was conducted in a greenhouse with an average daytime temperature of 30 °C and nighttime temperature of 26 °C, simulating ambient tropical air conditions. Bacterial suspensions (OD_600_ = 0.5) were applied at the base of each seedling (1 mL per plant). The inoculant consisted of 1 mL of sterile distilled water containing the bacterial suspension adjusted to OD₆₀₀ = 0.5. This OD value corresponded to approximately 1 × 10⁸ CFU/mL, based on serial dilution and plate counting. The inoculant was applied once at transplanting. Control plants received 1 mL of sterile distilled water without bacteria to ensure that the suspension matrix did not influence plant growth. All lettuce seedlings, including those in all four treatment groups (i–iv), were watered daily with an equal amount of nutrient solution containing 1.00 mM CaCl_2_·2H_2_O, 1.00 µM MnSO_4_·H_2_O, 0.50 mM KH_2_PO_4_, 0.25 mM MgSO_4_·7H_2_O, 0.30 µM H_3_BO_3_, 0.25 mM K_2_SO_4_, 0.50 µM Zn*S*O_4_·H_2_O, 0.01 µM NaMoO_2_·H_2_O, 0.20 µM CuSO_4_·5H_2_O, 0.01 µM CoSO_4_·7H_2_O, and 10.00 µM Fe citrate to ensure uniform nutrient availability across treatment. Maintenance of moisture content and watering of the plants were performed manually every day. All other culture practices were conducted in a controlled greenhouse environment at MJIIT, UTM KL, Kuala Lumpur, Malaysia, following standard protocols for temperate crop cultivation. These included manual watering, pest monitoring, and ambient temperature maintenance (average 27 ± 2 °C), without the use of soil cooling systems. One month after the plants were planted, dry weight, height, number of leaves, and root length were measured. Soil samples were taken from each experimental area, with three subsamples for each pot.

### Total nitrogen measurement in soils

The total nitrogen of the soil was evaluated after harvesting (for inoculation of bacteria) using the Kjeldahl method in triplicate according to the standard protocol (MS 417: Part 3: 1994). Briefly, 0.25 g of soil sample was digested with concentrated sulfuric acid in the presence of a catalyst mixture to convert organic nitrogen into ammonium. Following digestion, the reaction mixture was rendered alkaline with sodium hydroxide and subjected to steam distillation to release ammonia, which was trapped in hydrochloric acid. The amount of ammonia was subsequently quantified by titration using sodium hydroxide, with the endpoint determined using a mixed indicator. Total nitrogen content was calculated according to the standard Kjeldahl equation. Each nitrogen analysis was performed from three independent soil samples per treatment (*n* = 3).

### Statistical analysis

All data were tested for normality using the Shapiro-Wilk test and for homogeneity of variances using Levene’s test. For comparisons involving more than two treatments, one-way analysis of variance (ANOVA) was performed, followed by Tukey’s honestly significant difference (HSD) post-hoc test (*p* < 0.05). All analyses were conducted using IBM SPSS Statistics (version 23). Data are presented as mean ± standard deviation (SD), and different lowercase letters in the tables indicate significant differences among treatments (*p* < 0.05).

## Conclusion

The findings of this study underscore the relevance of exploring strategies to support temperate crop cultivation under tropical conditions and highlight the potential of indigenous soil bacteria as plant growth-promoting candidates. The bacterial isolates examined were associated with improved lettuce growth and changes in total soil nitrogen under controlled greenhouse conditions; however, these effects cannot be conclusively attributed to biological nitrogen fixation based on the screening approaches employed. The results suggest that bacteria exhibiting putative diazotrophic traits may contribute to plant performance through multiple mechanisms, which warrant further investigation. Future research should focus on functional validation of nitrogen fixation activity using direct assays, as well as the evaluation of additional plant growth-promoting traits and performance across different crops and environmental conditions. Such studies will be essential to assess the broader applicability of these bacteria in the context of sustainable agriculture under changing climatic conditions.

## Supplementary Information

Below is the link to the electronic supplementary material.

Supplementary Material 1

## Data Availability

The datasets generated and/or analyzed during the current study are available in the NCBI GenBank repository, under BioProject accession number PRJNA825673 (BioSamples: SAMN27553938 and SAMN27553940; GenBank assemblies: JALPSX000000000 and JALPSZ000000000). All other relevant data are included in the article and Supplementary Information.
