# Supplementary material for: Eco-functional deployment of indigenous nitrogen-fixing microbes to enable temperate crop cultivation in tropical climates
Source: Sci Rep. 2026 Mar 6;16:12396. doi: 10.1038/s41598-026-43406-x (PMC13084045; doi:10.1038/s41598-026-43406-x)
Supplement: Supplementary file 1 — Supplementary Material 1 [file 41598_2026_43406_MOESM1_ESM.docx]

**Supplementary Information:**

**Eco-Functional Deployment of Indigenous Nitrogen-Fixing Microbes to Enable Temperate Crop Cultivation in Tropical Climates**

Shazwana Shaárani, Nurul Syazwani Ahmad Sabri, Fatimah Azizah Riyadi , Siti Noor Fitriah Azizan, Fazrena Nadia Md Akhir, Nor’azizi Othman, and Hirofumi Hara**^*^**

**Supplementary information content:**

Figure S1. Growth of isolated *Agromyces* sp. C10 (left) and *Bacillus* sp. C21 (right) in nitrogen-free media

Figure S2. Representative image of *Agromyces* sp. C10 nitrogen fixation activity indicated by color change of agar from yellow to blue.

Figure S3. Lettuce growth with or without fertilizer and inoculation with *Agromyces* sp. C10 or *Bacillus* sp. C21.

Table S1. TBLASTN homology search for the identification of known nitrogen fixation gene in the draft genome sequence of *Agromyces* sp. C10 and *Bacillus* sp. C21.

Table S2. Classification of predicted proteins in subsystem features and their distribution in various functional groups shown in *Agromyces* sp. C10.

Table S3. Classification of predicted proteins in subsystem features and their distribution in various functional groups shown in *Bacillus* sp. C21.

Table S4. Annotated genes related to nitrogen-fixing/metabolism in *Agromyces* sp. C10.

Table S5. Annotated genes related to nitrogen-fixing/metabolism in *Bacillus* sp. C21.

Table S6. Primer sequences and PCR amplification conditions used for screening of nitrogen fixation-related genes


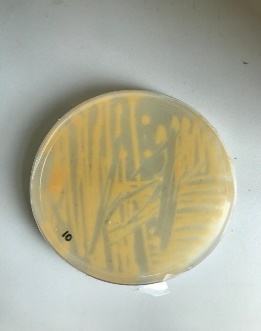

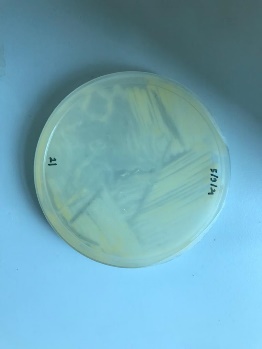


**Figure S1.** Growth of isolated *Agromyces* sp. C10 (left) and *Bacillus* sp. C21 (right) in nitrogen free media.


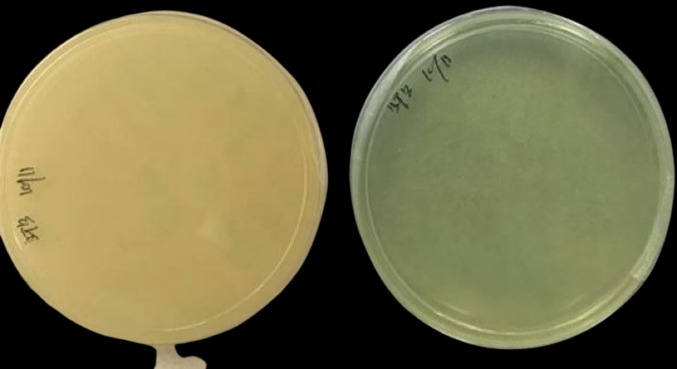


**Figure S2.** Representative image of *Agromyces* sp. C10 nitrogen fixation activity indicated by color change of agar from yellow to blue.


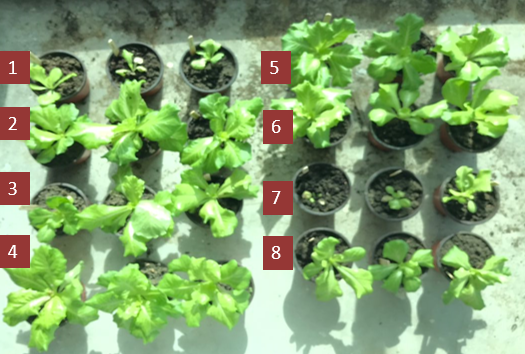

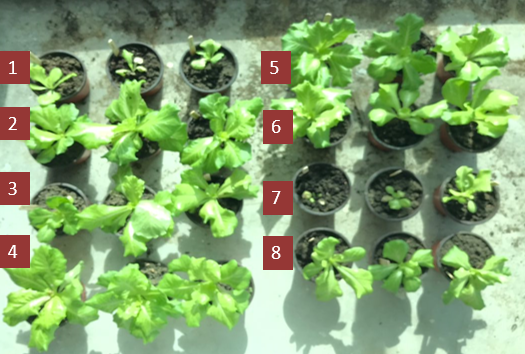

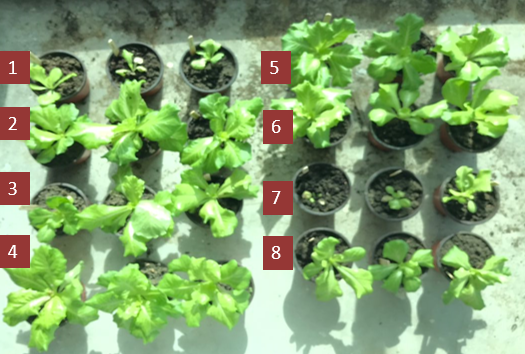


**Figure S3.** Lettuce growth with or without fertilizer and inoculation with *Agromyces* sp. C10 or *Bacillus* sp. C21.

Lane 1: Control (no fertilization and no inoculation of bacteria)

Lane 2: Fertilizer

Lane 3: *Agromyces* sp. C10

Lane 4: Fertilizer + *Agromyces* sp. C10

Lane 5: *Bacillus* sp. C21

Lane 6: Fertilizer + *Bacillus* sp. C21

**Table S1. TBLASTN homology search for the identification of known nitrogen fixation gene in the draft genome sequence of Agromyces sp. C10 and Bacillus sp. C21**

| **Strain** | **Draft genome analysis** | | | | |
| --- | --- | --- | --- | --- | --- |
|  | **Characterized in** | **Gene** | **Length amino acid** | **TBLASTN Identity** | **Score (Bits)** |
| *Agromyces* sp. C10 | *Bradyrhizobium sp.* AT1 | *anf* | 451 | 39% (176/452) | 253 |
|  | *Azotobacter*  *chroococcum* mcd 1 | *vnf* | 290 | 56% (18/32) | 239 |
|  | *Streptococcus pneumoniae* | *nifF* | 227 | 33% (64/192) | 99.8 |
|  | *Azotobacter vinelandii* | *nifD* | 492 | 21% (13/37) | 20.7 |
|  | - | *nifK* | No hits found | | |
|  | *Bacillus subtilis* (strain 168) | *glnR* | 135 | 38% (26/69) | 57.4 |
| *Bacillus* sp. C21 | *Bradyrhizobium sp.* AT1 | *anf* | 451 | 39% (174/447) | 296 |
|  | *Azotobacter*  *chroococcum* mcd 1 | *vnf* | 290 | 22% (56/250) | 45.8 |
|  | *Streptococcus pneumoniae* | *nifF* | 227 | 38% (78/208) | 134 |
|  | *Azotobacter vinelandii* | *nifD* | 492 | 37% (14/38) | 27.3 |
|  | *Bacillus subtilis* (strain 168) | *glnR* | 135 | 100(135/135) | 277 |
|  | - | *nifK* | No hits found | | |

**Table S2. Classification of predicted proteins in subsystem features and their distribution in various functional groups shown in *Agromyces* sp. C10**

| **Class** | **Number of genes** | **Description** |
| --- | --- | --- |
| J | 151 | Translation, ribosomal structure and biogenesis |
| A | - | RNA processing and modification |
| K | 237 | Transcription |
| L | 126 | Replication, recombination and repair |
| B | 1 | Chromatin structure and dynamics |
| D | 36 | Cell cycle control, cell division, chromosome partitioning |
| V | 55 | Defense mechanisms |
| T | 90 | Signal transduction mechanisms |
| M | 133 | Cell wall/membrane/envelope biogenesis |
| N | 7 | Cell motility |
| Z | - | Cytoskeleton |
| U | 26 | Intracellular trafficking, secretion, and vesicular transport |
| O | 93 | Posttranslational modification, protein turnover, chaperons |
| C | 164 | Energy production and conversion |
| G | 230 | Carbohydrate transport and metabolism |
| E | 316 | Amino acid transport and metabolism |
| F | 88 | Nucleotide transport and metabolism |
| H | 108 | Coenzyme transport and metabolism |
| I | 110 | Lipid transport and metabolism |
| P | 107 | Inorganic ion transport and metabolism |
| Q | 40 | Secondary metabolites biosynthesis, transport and catabolism |
| R | - | General function prediction only |
| S | 570 | Function unknown |
| - | 216 | Not in COGs |

**Table S3. Classification of predicted proteins in subsystem features and their distribution in various functional groups shown in *Bacillus* sp. C21**

| **Class** | **Number of genes** | **Description** |
| --- | --- | --- |
| J | 174 | Translation, ribosomal structure and biogenesis |
| A | 2 | RNA processing and modification |
| K | 329 | Transcription |
| L | 136 | Replication, recombination and repair |
| B | 1 | Chromatin structure and dynamics |
| D | 43 | Cell cycle control, cell division, chromosome partitioning |
| V | 56 | Defense mechanisms |
| T | 117 | Signal transduction mechanisms |
| M | 200 | Cell wall/membrane/envelope biogenesis |
| N | 59 | Cell motility |
| Z | - | Cytoskeleton |
| U | 32 | Intracellular trafficking, secretion, and vesicular transport |
| O | 87 | Posttranslational modification, protein turnover, chaperons |
| C | 204 | Energy production and conversion |
| G | 225 | Carbohydrate transport and metabolism |
| E | 354 | Amino acid transport and metabolism |
| F | 97 | Nucleotide transport and metabolism |
| H | 116 | Coenzyme transport and metabolism |
| I | 132 | Lipid transport and metabolism |
| P | 199 | Inorganic ion transport and metabolism |
| Q | 63 | Secondary metabolites biosynthesis, transport and catabolism |
| R | - | General function prediction only |
| S | 982 | Function unknown |
| - | 202 | Not in COGs |

**Table S4. Annotated genes related to nitrogen-fixing/metabolism in *Agromyces* sp. C10**

| **Feature ID/Gene position** | **Function** | **Size (amino acid)** | **Organism** | **Query cover** | **E-value** | **BLASTP Identity** | **Bit score** | **Best match accession no.** |
| --- | --- | --- | --- | --- | --- | --- | --- | --- |
| fig\|6666666.909669.peg.2917 | Nitrogen metabolism regulator GlnR, OmpR family | 229 | *Agromyces flavus* | 96% | 1E-157 | 62% | 269 | WP_092675436.1 |
| fig\|6666666.909669.peg.637 | Glutamate N-acetyltransferase (EC 2.3.1.35) @ N-acetylglutamate synthase (EC 2.3.1.1) | 385 | Unclassified Agromyces | 100% | 0 | 63% | 459 | WP_067949185.1 |
| fig\|6666666.909669.peg.1645 | Two-component transcriptional response regulator, OmpR family | 218 | *Agromyces* sp. Marseille-P2726 | 81% | 0 | 46% | 184 | WP_173922746.1 |
| fig\|6666666.909669.peg.2708 | Transcriptor LysR family | 321 | *Agromyces* sp. NDB4Y10 | 99% | 1E-147 | 28% | 76 | WP_204392161.1 |
| fig\|6666666.909669.peg.8 | Transcriptor LysR family | 295 | *Agromyces* sp. NDB4Y10 | 99% | 0 | 41% | 181 | WP_067946053.1 |
| fig\|6666666.909669.peg.85 | Transcriptor LysR family | 313 | *Agromyces* sp. NDB4Y10 | 98% | 0 | 40% | 190 | WP_067946270.1 |
| fig\|6666666.909669.peg.348 | Transcriptor LysR family | 330 | *Agromyces kandeliae* | 79% | 3E-170 | 22% | 81 | WP_154346342.1 |
| fig\|6666666.909669.peg.473 | Transcriptor LysR family | 353 | *Agromyces* sp. NDB4Y10 | 99% | 0 | 30% | 116 | KZE94557.1 |
| fig\|6666666.909669.peg.785 | Transcriptor LysR family | 310 | *Agromyces* sp. NDB4Y10 | 100% | 0 | 30% | 74 | WP_067945913.1 |
| fig\|6666666.909669.peg.1358 | Transcriptional regulator ArgP, LysR family | 301 | *Agromyces* sp. NDB4Y10 | 99% | 0 | 47% | 233 | WP_067950765.1 |
| fig\|6666666.909669.peg.1510 | Transcriptor LysR family | 310 | *Agromyces* sp. NDB4Y10 | 98% | 0 | 33% | 138 | WP_067946040.1 |
| fig\|6666666.909669.peg.2161 | Transcriptor LysR family | 228 | *Agromyces* sp. NDB4Y10 | 99% | 0 | 29% | 56 | WP_067946546.1 |

**Table S5. Annotated genes related to nitrogen-fixing/metabolism in *Bacillus* sp. C21**

| **Feature ID/Gene position** | **Function** | **Size (amino acid)** | **Organism** | **Query cover (%)** | **E-value** | **BLASTP Identity** | **Bit score** | **Best match accession no.** |
| --- | --- | --- | --- | --- | --- | --- | --- | --- |
| fig\|6666666.910181.peg.995 | Nitrogen regulation protein NR(I), GlnG (=NtrC) | 331 | *Bacillus subtilis* QB928 | 100 | 0 | 99% | 651 | AFQ58124.1 |
| fig\|6666666.910181.peg.1164 | Glutamate synthase operon transcriptional activator GltC, LysR family | 300 | *Bacillus* | 100 | 0 | 100% | 590 | WP_004399246.1 |
| fig\|6666666.910181.peg.438 | Transcriptional regulator, LysR family | 299 | *Bacillus subtilis subsp. subtilis str.* SC-8 | 100 | 0 | 99% | 578 | EHA31126.1 |
| fig\|6666666.910181.peg.1168 | Transcriptional regulator, LysR family | 285 | *Bacillus subtilis* | 100 | 0 | 99% | 558 | WP_029318093.1 |
| fig\|6666666.910181.peg.1250 | Transcriptional regulator, LysR family | 292 | *Bacillus* | 100 | 0 | 99% | 580 | WP_167408607.1 |
| fig\|6666666.910181.peg.1734 | LysR-family transcriptional regulator Bsu YwqM | 293 | *Bacillus* | 100 | 0 | 99% | 578 | WP_042975542.1 |
| fig\|6666666.910181.peg.3933 | Transcriptional regulator, LysR family | 324 | *Bacillus* | 100 | 0 | 99% | 628 | WP_029727059.1 |

**Table S6. Primer sequences and PCR amplification conditions used for screening of nitrogen fixation-related genes**

| **Target gene** | **Primer** | **Sequence (5'-3')** | **Annealing (°C)** | **PCR conditions** | **Reference** |
| --- | --- | --- | --- | --- | --- |
| nifH | PolF | TCGAYCCSAARGCBGACTC | 55 | 94 °C 1 min; 98 °C 10 s; 55 °C 1 min; 72 °C 2 min (30 cycles); final 72 °C 5 min | [63] |
| nifH | PolR | ATSGCCATCATYTCRCCGGA | 55 | 94 °C 1 min; 98 °C 10 s; 55 °C 1 min; 72 °C 2 min (30 cycles); final 72 °C 5 min | [63] |
| nifD | nifD-up | ATCATCGGTGACTACAAC | 52 | 94 °C 5 min; 94 °C 30 s; 52 °C 30 s; 72 °C 30 s (35 cycles); Last cycle, sample were maintained at annealing temperature for 5 min followed by 72 °C 10 min | [64] |
| nifD | nifD-do | ATCCATGTCGCGGCG AA | 52 | 94 °C 5 min; 94 °C 30 s; 52 °C 30 s; 72 °C 30 s (35 cycles); Last cycle, sample were maintained at annealing temperature for 5 min followed by 72 °C 10 min | [64] |
| nifK | nifHD-f | CAGGAAATCTACATCGTCATGTC | 63 | 94 °C 30 s; 92 °C 30 s; 63 °C 30 s; 72 °C 90 s (35 cycles); final 72 °C  5 min | [65] |
| nifK | nifD-r | TCCCANGARTGCATCTGRCGG | 63 | 94 °C 30 s; 92 °C 30 s; 63 °C 30 s; 72 °C 90 s (35 cycles); final 72 °C  5 min | [65] |
